# Modulating Role of Vitamins C and E against Transport-Induced Stress in Pullets during the Hot-Dry Conditions

**DOI:** 10.5402/2011/497138

**Published:** 2010-10-28

**Authors:** N. S. Minka, J. O. Ayo

**Affiliations:** ^1^College of Agriculture and Animal Science, Division of Agricultural Colleges, P. M. B 2134, Ahmadu Bello University, Mando, 2134 Kaduna, Nigeria; ^2^Department of Physiology and Pharmacology, Faculty of Veterinary Medicine, Ahmadu Bello University, 1082 Zaria, Nigeria

## Abstract

The modulating effects of ascorbic acid (AA), vitamin E (E), and a combination of AA and E (AA + E) against eight-hour road transportation stress were investigated in 120 pullets during the hot-dry season. The result obtained showed that handling, loading and transportation induced lymphopenia, neutrophilia, liveweight loss, and mortality, which was alleviated by oral administration of AA, E, and AA + E at doses of 60 mg, 30 mg, and 60 + 30 mg per kg bodyweight, respectively, 30 minutes before being loaded and transported. The meteorological conditions recorded during the study period were higher (*P* < .05) than the thermoneutral values established for chickens in the zone. In conclusion, the administration of vitamins AA, E, and AA + E, especially AA, ameliorated the risk of adverse effects of handling, loading, transportation, and thermal stress in pullets during the hot-dry season.

## 1. Introduction

The increase in demand for poultry meat and eggs has necessitated the establishment of new farms and slaughter houses away from their original places of production, and consequently, a considerable increase in poultry transportation arises [1, 2]. Results obtained from an increasing body of research have convincingly demonstrated that the very act of handling, crating, loading, and transportation of poultry has adverse effects on the welfare, health, and productivity of birds [1, 3, 4]. The establishment of a standard transport welfare animal order by many countries [5, 6] and its strict compliance by transporters did not eliminate completely the welfare problems encountered in transported food animals. Consequently, poultry farmers during transportation of birds still incur substantial economic loses [1, 7]. 

In general, there is a paucity of information on the responses of point-of-lay pullets belonging to Black Harco breed to long distance road transportation, and the methods of alleviating road transportation stress in poultry in the tropics with classically hot climatic conditions. Although road transportation stress cannot be eliminated completely in poultry, the applications of tranquilizers, electrolytes, and amino acids have been observed to alleviate the stress. Unfortunately, the residual effects of some of these drugs contravene legislative requirements for wholesome meat [5, 8, 9]. The need to seek for reliable, cheaper, easily administered, readily available, and nontoxic prophylactic agents to combat the adverse effects of road transportation stress without any negative effect has become paramount. 

Ascorbic acid (AA) and vitamin E (E) are known to have immunomodulating effects. They are used as therapeutic agents against many diseases, especially heat stress in humans [10, 11], poultry [2, 12, 13], and other livestock [14, 15]. The ameliorating effects of the vitamins are well manifested when the body AA or E is either overwhelmed or exhausted as a result of many stress factors that overtax the animal control systems [12]. Heterophil/lymphocyte ratio and survivability have been used as reliable welfare indices in evaluating the adaptability of animals to various stress factors [2, 12, 13, 16].

The aims of the present study were to investigate changes in blood parameters, liveweight loss, traumatic injury, and mortality encountered by Black Harco pullets during road transport and to suggest the use of antioxidant vitamins AA and E as an antistress.

## 2. Materials and Methods

### 2.1. Birds and Management

Three hundred and eighty (380) female pullets (Black Harco strain) obtained as day-old chicks were raised first in a brooding room and later in a standard deep litter poultry house till 18 weeks' old. They were fed certified standard feeds obtained from Feed Master Ltd., Kaduna, Nigeria, and water was provided *ad libitum*. 160 apparently healthy pullets of similar body weight (1.0 ± 0.1 kg) were selected randomly and partitioned separately from the rest birds within the same pen. These birds served as experimental birds. The birds were stocked at a rate of 0.25 m^2^/bird from 12 weeks old. After the journey, the pullets were unloaded into a standard deep litter poultry house partitioned into four chambers, which housed each group at a stocking rate of 0.25 m^2^/bird. For each chamber, two drinking (0.5 m length × 4 cm width each) and feeding (0.5 m × 10 cm each) troughs were provided. Deep litter system of management and noncrating of the birds during the journey were adopted in order to reduce the stress due to confinement, and also in compliance with the new EU council directive 99/74/EC to phase out the conventional layer cages by year 2012.

All necessary vaccinations, medications, and other related management practices were strictly adhered to as recommended by the National Veterinary Research Institute (NVRI), Jos, Nigeria.

### 2.2. Meteorological Data

The meteorological conditions of the study area inside and outside the pens before the experiment and at the new site where the birds were transported to were recorded for one week before and after the journey at 06:00, 13:00, and 18:00 hours. During the journey period, the ambient temperature (AT) and relative humidity (RH) were recorded both inside and outside of the vehicle. The meteorological data were recorded with the aid of a wet- and dry-bulb thermometer (Brannan, England)

### 2.3. Experimental Design

Food was withdrawn from the birds 8 hours before the journey, while water was withdrawn two hours before the journey as suggested by Metheringham and Hubrecht [1]. On transportation day, the birds were randomly divided by partitioning into four groups of 40 birds each. Each group of birds was identified by pasting an adhesive plaster on the wing of each bird. Birds in group I served as control pullets (control) and were administered orally 2 mL/kg body weight of drinking water. Group II pullets (AA) were administered orally ascorbic acid (AA) at a dose of 60 mg/kg body weight [2, 17] dissolved in sterile water. Group III (E) pullets were administered orally vitamin E at a dose of 30 mg/kg body weight [18], while group IV (AA + E) pullets were administered orally a combination of AA and E at the aforementioned doses, respectively. The administration of the vitamins and water was done by gentle feeding through the beak of each bird with minimal stress [2]. 

Ten birds from each group were colour marked and quickly bled just before the administration of the vitamins and the process of loading to obtain baseline values. Thirty minutes after loading, and before the start of driving, another set of 10 birds from each group were bled to evaluate the stresses imposed by handling and loading procedures. The 30 minutes lapse between preloading and postloading sampling was allowed because it takes 20–30 minutes for cortisol concentration to peak in circulation after imposing a stressor [19]. Thereafter, no further blood sampling was done on these groups of birds again. Immediately after the journey when the birds was unloaded another set of 10 birds from each group were bled to evaluate the stress due to transportation. Three hours posttransportation, the remainig 10 birds from each group that were not bled before were bled. Finally, three days posttransportation period, 10 birds were selected randomly from each group and bled. This design was adopted in order to eliminate carrying-over effect of repeated handling and blood sampling of the same birds.

### 2.4. Vehicle Design and Journey Duration

The vehicle used for the journey was a modified Bedford bus (made in England) with an internal floor dimension of 4 × 1.2 m. The vehicle had four windows made of louvers on both side and two from the rear side of the vehicle. The windows (50 × 35 cm each) were located 800 cm from the floor level of the bus which regulated and provided adequate ventilation. The top roof of the bus was made up of a metal roof stock-pilled with heat absorbable foam materials from the inside and covered with a thick polythene ceiling. The floor was made up of metal and was covered with rough chopped saw dust, and a thick locally woven nonslippery rubber mat was placed on top. The floor was partitioned diagonally into two equal left and right chambers. Each chamber was stocked with 160 pullets consisting of 40 birds selected from each group. This arrangement was done in order to provide a similar condition for all the birds during the journey [20]. The pullets were stocked at a rate of 0.16 m^2^/bird. The vehicle travelled on a tarred road for 8 hours, covering a distance of 360 km at an average speed of 45 km/h.

### 2.5. Blood Sampling and Analysis

The blood sampling was done using disposable vacutainer needles. About two drops of blood was collected from the wing vein of each bird as quickly as possible. From each sample, two blood smears were made on microscopic slides immediately after the blood was collected. The blood smears were dried and stained with Camco Quick Stain II, Buffered Differential Wright Giemsa Stain (Bayer Corp., Diagnostic Division, Elkhart, IN). Heterophil : lymphocyte (H/L) ratios were determined by using a 100/1.25 oil immersion objective, and 100 cells per slide were counted using the straight edge method [21]. The number of heterophils was divided by the total number of lymphocytes to obtain the H/L ratio.

### 2.6. Detection of Traumatic Injuries

Detection of injuries was done as earlier described by Smirnov et al. [22] and Minka and Ayo [23]. Briefly, during the loading and unloading of the bird, each bird was quickly examined physically by palpation and raising the feathers with one hand against the direction of the feathers which paved way to examine the underlying skin for any injury or abnormality. Birds discovered with injuries or abnormalities during loading were not transported. Birds that sustained conspicuous injuries during the journey were calmly restrained and the injured areas were cleaned with cotton wool soaked with an antiseptic, after which an adhesive plaster marked with the nature of injury was pasted on the injured site. These birds were excluded from further blood sampling throughout the study period. Injuries recorded were defined as described by Shakalov et al. [24] and their locations and severity categorized as severe (fracture, dislocation, and wounds), mild (contusions, abrasion, and laceration), and no injury at all [23]. The number of pullets found dead before, during and after transportation period was recorded.

### 2.7. Body Weight Measurement

The body weight of 20 birds from each group was recorded before, immediately after and three days posttransportation. The liveweight was recorded using a digital weight scale (Phillip Harris, England).

The handling, loading and transportation of the pullets were done humanely as recommended by the animal transport welfare order and the code for practice for the land transport of poultry [6, 25].

### 2.8. Statistical Analysis

The results were expressed as mean ± S.E.M, and analysis of variance was used to separate means and result comparison to give an indication of any differences between the groups of the pullets. Values of *P* < .05 were considered significant.

## 3. Results

### 3.1. Meteorological Data

The meteorological conditions during the study period are shown in Table 1. The AT values recorded outside the pen before and posttransportation were significantly (*P* < .05) higher than the corresponding values recorded inside the pen. There were no significant (*P* > .05) differences in the AT values before and three days posttransportation at the new site. The mean AT and RH recorded inside the vehicle during the journey were significantly (*P* < .05) higher than the corresponding values recorded outside the vehicle.

### 3.2. Mortality

Mortality of 20% was recorded in control pullets, 0% in pullets administered AA, 2.5% in E, and 5% in pullets administered AA + E. The overall percent mortality in control pullets was higher (*P* < .05) than the values recorded for AA, E, and AA + E pullets (Table 2). 80% of the mortality which occurred in the control pullets was without any signs of injury, while 20% had different types of injuries.

### 3.3. Heterophil/Lymphocytes Ratios

The H/L ratios of the pullets are shown in Table 2. The pretransport H/L ratios in all the pullets were not different (*P* > .05) from one another. The H/L ratio obtained in AA pullets 30 minutes after loading just before the start of the journey was not different (*P* > .05) from the pre-loading value. In control, E, and AA+E pullets the post-loading H/L ratios were significantly (*P* < .05) higher than the pre-transport and corresponding post-transport H/L ratio values recorded in AA pullets. The H/L ratio obtained immediately and three hours posttransportation period in AA, E and AA + E pullets were not statistically different (*P* > .05) between the groups, but the values were significantly lower (*P* < .05) than those recorded in control pullets. Three days posttransportation, the H/L ratio in control pullets was not statistically different (*P* > .05) from the value recorded from pullets administered AA, E, or AA + E, and also from the corresponding pre-transport values.

### 3.4. Injury

The percent number of injured birds, and severity and distribution of the injuries according to affected body parts are shown in Table 3. There was no significant difference in the values of injuries recorded in control, AA, E, and AA + E pullets. In the overall, more injuries were sustained on the head and neck regions than any other part of the body. Severe injures observed were mostly wounds and dislocations. No fracture was recorded. The most common type of injury sustained by the pullets in all the groups was mild (lacerations).

### 3.5. Liveweight of Pullets

The liveweights of the pullets immediately after the journey were lower than the pre-transport values in all the groups. However, in control, E, and AA + E pullets, the percent liveweight loss was significantly (*P* < .05) higher than the corresponding posttransportation value recorded for AA pullets. Three days posttransportation period, only AA pullets regained their pre-transportation liveweight values (Table 4).

## 4. Discussion

The meteorological data recorded during the study period, especially during the afternoon hours of the day inside and outside the poultry house and in the vehicle during the journey period were outside the thermoneutral zone of 22.0 to 28.5°C, established for chickens predominantly reared in the tropics [26]. The wide fluctuation in AT and RH occurring during the day, especially during the journey period, when other transportation stress factors acted concomitantly on the pullets, aggravated the adverse effects of road transportation on the pullets. Studies have shown that it is not only excessively high temperatures that affect birds, but, also the fluctuations of the AT and RH [5, 27]. The meteorological result obtained in the present study did not favour transportation of the pullets.

The significant (*P* < .01) increase in H/L ratio recorded in control pullets immediately after, three hours, and three days posttransportation period showed that the immune system of the birds were compromised as a result of transportation stress up to the third day posttransportation. The increase in H/L ratio obtained in the present study agrees with the findings of several studies which demonstrated that stress conditions, especially thermal stress, decrease both humoral and cellular immune responses [16, 23, 28], resulting into increase in H/L ratio. Furthermore, the lower H/L ratio obtained 30 minutes after loading, just before the start of the journey in AA pullets showed, for the first time, that AA abolished the stresses induced by handling and loading of pullets. Handling and loading of livestock are more stressful to transported birds than the journey itself [29]. The H/L ratio has been accepted as the most reliable index for determining long-term effect of various stressors in poultry [2, 4, 30] and other livestock [15, 19]. The lower values of H/L ratio recorded in pullets administered AA, E, and AA + E posttransportation suggested that the vitamins reduced or eliminated the adverse effect of road transportation stress on the immune system of the pullets. Both AA and E are known to be chain-breaking antioxidants, involved in the prevention and restriction of free radical chain formation and propagation, and consequently, protecting the blood cells from oxidative damage [31–33]. Similarly, AA and vitamin E are shown to inhibit the release of corticosteroid, a hormone known to destroy immune cells [11, 33, 34]. 

The 20% mortality recorded in control pullets compared to 0%, 2.5%, and 5% recorded in pullets administered AA, E and AA + E, respectively, showed that road transportation of pullets during the hot-dry period causes economic losses, especially in birds not administered with antioxidant vitamins. Similar economic loses due to road transport stress in broilers were reported in Britain, where about 600,000 broilers died annually as a result of transportation to slaughter houses [1]. Although the proximate cause of death in the present study was not investigated, it is suggestive that heat stress was responsible. The present finding agreed with those of Bayliss and Hinton [7], who recorded 40% of deaths in transported broilers as a result of heat stress. The low mortality recorded in pullets administered AA or E suggested that the mortality often encountered during road transportation could be reduced to minimum or eliminated completely by the administration of antioxidant vitamins prior to transportation. This finding is of economic importance for poultry industries because the antioxidant vitamins used in the present study were nontoxic, relatively cheap, and readily available and do not have any residual effects even at higher doses [11]. The present results agree with those of Hicks [35], Gill et al. [36], and Nockels [28], who showed that AA and vitamin E reduce the duration of sickness, percent of morbidity and mortality in animals. 

The results of the injuries indicated that transportation was associated with injuries, some of which were responsible for transport mortality. The number of birds found dead without any sign of injury was higher (*P* < .05) than those with injuries, which indicated that heat stress was a major factor responsible for transport mortality in the pullets. The higher (*P* < .05) percentage of injuries located on the head and neck regions was as a result of fighting and pecking behaviours. The results of injuries and fractures obtained in the present study were lower than those recorded in broilers by Bayliss and Hinton [7] and Metheringham and Hubrecht [1], who reported that about 35% injuries been responsible for dead-on-arrival of broilers in slaughtering plants. Even though there was no significant (*P* > .05) effect of antioxidant vitamins on injuries sustained, the higher values of injuries recorded in AA and E pullets may be as a result of increased excitation caused by the administration of the vitamins [14, 17], especially AA which might have increased the pecking and fighting behaviours of the birds. This requires further investigation.

The loss in liveweight posttransportation was as a result of prolong food and water deprivation, increased muscle depletion, and elimination of the gut content brought about by increase in excitation of the nervous system and also due to heavy water loss, probably caused by panting. The insignificant weight loss and the restoration of the initial liveweight just 3 days posttransportation in AA pullets were evidence that AA reduces liveweight losses often encountered posttransportation period. The finding agreed with the results obtained in transported goats administered with AA [15].

The overall result showed that the combination of AA and E had less ameliorating effect on the transported pullets compared to when AA was administered singly. The present result contradicts the overwhelming evidence of the synergistic role of AA and E, and also the sparing effect of AA on vitamin E [11, 17, 33]. This may require more investigation.

 In conclusion, the administration of antioxidant vitamins AA, E, and AA + E, especially AA, alleviated the risk of adverse effects of road transportation stress in pullets during the hot-dry season.

## Figures and Tables

**Table 1:** Meteorological data during the study period.

Period	Ambient temperature (°C, mean ± SEM)				
	Mean	Maximum	Minimum	Range	Relative humidity (%)
Pre-transport:					
Outside pen	37.2 ± 1.1^a^	39.7	27.2	12.5	58 ± 6.4^a^
Inside pen	34.3 ± 0.9^b^	36.4	28.4	8.0	56 ± 2.4^a^
During transport:					
Outside vehicle	37.2 ± 0.2^a^	39.6	29.4	10.2	54 ± 4.2^a^
Inside vehicle	38.2 ± 0.7^a^	38.7	30.5	8.2	62 ± 8.4^b^
Post-transport:					
Outside pen	37.0 ± 0.9^a^	39.0	24.8	15.1	51 ± 6.9^a^
Inside pen	35.6 ± 0.4^b^	37.4	28.6	8.8	54 ± 8.4^a^

For each parameter, mean values with different superscript alphabets along the same column are significantly (*P* < .05) different.

**Table 2:** Effect of road transportation, ascorbic acid (AA), vitamin E and a combination of AA + E on the number of mortality and heterophil/lymphocyte ratio of pullets.

Parameters	Pullets			
	Control	AA	E	AA + E
Mortality (number of pullets):				
Before transportation	0	0	0	0
During transportation	5	0	0	2
Three hours posttransportation	2	0	1	0
Three days post-transport	1	0	0	0
Overall mortality	8	0	1	2
Percent mortality (%)	20	0	2.5	5
Heterophil/lymphocyte Ratio:				
Before transportation	0.40 ± 0.8^a^	0.41 ± 0.6^a^	0.39 ± 0.7^a^	0.40 ± 0.7^a^
30 minutes after loading	0.63 ± 1.1^a^	0.43 ± 0.4^b^	0.60 ± 0.9^a^	0.58 ± 0.3^a^
Immediately after transportation	0.62 ± 0.7^a^	0.49 ± 0.2^b^	0.50 ± 0.8^b^	0.52 ± 0.2^b^
Three hours posttransportation	0.60 ± 0.2^a^	0.46 ± 0.9^b^	0.48 ± 1.0^b^	0.47 ± 07^b^
Three days posttransportation	0.42 ± 0.2^a^	0.40 ± 0.8^a^	0.41 ± 0.7^a^	0.39 ± 0.7^a^

Mean values with different superscript alphabets along the same row are significantly (*P* < .05) different.

**Table 3:** Effects of ascorbic acid (AA), vitamin E, and a combination of AA + E on the percent number, severity, and location of injuries sustained by the pullets during the journey.

Parameters	Control (*n* = 40)	AA (*n* = 40)	E (*n* = 40)	AA + E (*n* = 40)
Number of injured birds (%)	22.1 ± 1.2	24.5 ± 1.8	23.0 ± 2.0	22.5 ± 1.4
Severity of injuries (%):				
Severe	4.4 ± 1.3	5.2 ± 2.0	4.8 ± 2.7	4.5 ± 1.9
Mild	19.5 ± 2.6	20.0 ± 3.7	21.0 ± 2.1	18.7 ± 2.9
No injury	76.0 ± 3.4	75.2 ± 3.0	74.5 ± 2.9	76.8 ± 3.8
Location of injury (%):				
Head	10.0 ± 1.5	15 ± 1.3	12.0 ± 0.9	8.0 ± 1.4
Neck	7.0 ± 0.4	6.0 ± 1.0	9 ± 1.1	7.0 ± 0.9
Thorax/Abdomen	2.0 ± 1.0	2.0 ± 2.0	3.0 ± 1.6	4.0 ± 1.2
Wing	4.0 ± 0.9	3.0 ± 1.2	2.0 ± 1.7	4.0 ± 2.0
Limb	3.0 ± 1.0	6.0 ± 2.0	5.0 ± 2.3	4.0 ± 1.8

Mean values along the same row are not significantly (*P* > .05) different.

**Table 4:** Effects of ascorbic acid (AA), vitamin E and a combination of AA + E and road transportation stress on liveweights of transported pullets (kg, mean ± SEM).

Pullets	*n*	Before transport	After transportation		Percent liveweight difference (%)	
			8-hours	3-days	After 8 hours	After 3 days
Control	20	1.05 ± 0.8^a^	0.80 ± 0.1	0.89 ± 0.2	−23.8^a^	−15.2^a^
AA Pullets	20	1.03 ± 0.2^a^	1.02 ± 0.3	1.09 ± 0.6	−0.9^b^	+5.8^b^
E Pullets	20	1.06 ± 0.7^a^	0.91 ± 0.3	1.01 ± 0.6	−14.2^c^	−4.7^c^
AA + E Pullets	20	1.05 ± 0.3^a^	0.89 ± 0.6	1.03 ± 0.4	−15.2^c^	−1.9^d^

Values with different superscript alphabets along the same column are significantly (*P* < .05) different.
